# How leaders' mindfulness in communication mitigates employees' knowledge hiding behavior: the underlying mechanisms

**DOI:** 10.3389/fpsyg.2026.1880746

**Published:** 2026-09-11

**Authors:** Chaoran Li, Sijin Du, Jianjun Wang

**Affiliations:** 1 Business School, University of New South Wales, Sydney City, NSW, Australia; 2 School of Management, Xiamen University, Xiamen City, Fujian Province, China; 3 Office of Human Resources, Taizhou Polytechnic College, Taizhou City, Jiangsu Province, China; 4 College of Finance and Economics, Qinghai University, Xining City, Qinghai Province, China; 5 School of Economics and Management, BaoShan University, BaoShan City, Yunnan Province, China

**Keywords:** employee mindfulness, employees' knowledge hiding behavior, interpersonal trust, leader mindfulness in communication, organizational justice

## Abstract

Knowledge hiding occurs when employees deliberately withhold knowledge requested by others, potentially hindering knowledge exchange and collaboration. Drawing on social exchange theory and social information processing theory, this study examines how and when leaders' mindfulness in communication mitigates employees' knowledge hiding behavior. We propose a moderated model with two serial mediation paths, in which leaders' mindfulness in communication enhances employees' perceived organizational support, which subsequently reduces knowledge hiding through increased organizational justice and strengthened interpersonal trust, respectively. We further examine employee mindfulness as a boundary condition that amplifies the positive relationship between leaders' mindfulness in communication and perceived organizational support. Study 1 used a three-wave survey of 652 employees from Chinese technology firms to test the proposed model. Study 2 employed a four-week mindfulness training intervention involving 180 leaders and 540 employees to provide supplementary evidence for the intervention effect. The results showed that leaders' mindfulness in communication was negatively associated with employees' knowledge hiding through two pathways: perceived organizational support and organizational justice, and perceived organizational support and interpersonal trust. Employee mindfulness strengthened the relationship between leaders' mindfulness in communication and perceived organizational support. The intervention study further showed that training both leaders and employees led to greater reductions in knowledge hiding than training leaders alone or providing no mindfulness training. These findings extend research on knowledge hiding and workplace mindfulness by integrating exchange-based mechanisms with social information-processing perspectives, and they offer a practical intervention approach for promoting supportive communication and reducing defensive knowledge behaviors in organizations.

## 1 Introduction

Knowledge has become a central source of organizational adaptability and innovation, but it does not flow automatically simply because organizations need it (Nonaka, 1994; Wang and Noe, 2010). In many workplaces, valuable expertise, experience, and problem-solving insights are embedded in individual employees, who must decide whether sharing what they know will benefit them or put them at a disadvantage. When employees perceive that sharing knowledge may weaken their uniqueness, reduce their bargaining power, or expose them to unfair use of their contributions, they may choose to protect this resource rather than disclose it. This intentional withholding or concealment of knowledge requested by others is known as employees' knowledge hiding behavior (Connelly et al., 2012). Although employees' knowledge hiding behavior may appear to be a rational self-protection strategy for individual knowledge holders, its collective consequences are costly. Once employees' knowledge hiding behavior spreads within teams, it disrupts knowledge flow, weakens collaboration, and undermines organizational learning and innovation (Cerne et al., 2014; Connelly et al., 2019). Therefore, understanding how organizations can reduce employees' motivation to engage in employees' knowledge hiding behavior has become an important issue in organizational psychology and knowledge management.

Prior research has shown that leadership plays a meaningful role in shaping employees' knowledge behaviors (Singh, 2019). For example, ethical leadership and transformational leadership may reduce knowledge hiding by creating moral norms, supportive climates, or motivational conditions (Abdullah et al., 2019; Lei et al., 2022). However, relying primarily on broad leadership styles creates a practical challenge. Leadership styles are relatively stable and often difficult to change quickly, whereas organizations usually select and evaluate leaders based on competence, resources, and performance rather than on whether they already possess a specific leadership style. This creates a gap between what research recommends and what organizations can realistically implement. Rather than asking organizations to replace leaders or transform their entire leadership style, a more scalable approach is to identify concrete, trainable leadership behaviors that can reduce employees' defensive motives for hiding knowledge.

Leaders' mindfulness in communication offers such a possibility. It refers to leaders' attentive listening, non-judgmental acceptance, and empathic responding during interpersonal communication (Arendt et al., 2019). Unlike broad leadership styles, mindful communication is specific, observable, and trainable. When leaders communicate mindfully, they signal that employees' voices are heard, their concerns are taken seriously, and their contributions are valued. These communication cues may be especially important in knowledge-sharing contexts, where employees often weigh the potential benefits of helping others against the potential loss of personal advantage. By reducing perceived relational risk, leaders' mindfulness in communication may make knowledge sharing feel less threatening and offer a practical relational approach to reducing employees' knowledge hiding behavior.

To explain this process, we integrate social exchange theory and social information processing theory. Social exchange theory helps explain why supportive, fair, and trustworthy exchanges reduce employees' motivation to engage in employees' knowledge hiding behavior (Cropanzano and Mitchell, 2005), whereas social information processing theory helps explain how employees interpret leaders' mindful communication and why employee mindfulness may shape this interpretation. Together, these theories allow us to examine both the motivational mechanisms and the interpretive boundary condition through which leaders' mindfulness in communication reduces employees' knowledge hiding behavior.

Based on this integrated theoretical logic, we propose a moderated model with two serial mediation paths. Specifically, we argue that leaders' mindfulness in communication enhances employees' perceived organizational support, which subsequently reduces employees' knowledge hiding behavior by increasing organizational justice and strengthening interpersonal trust (Rhoades and Eisenberger, 2002). We further propose that employee mindfulness strengthens the positive relationship between leaders' mindfulness in communication and perceived organizational support (Glomb et al., 2011). This model positions perceived organizational support as a central psychological bridge, organizational justice and interpersonal trust as two parallel downstream mechanisms, and employee mindfulness as a boundary condition that shapes employees' interpretation of leaders' communication.

**Figure 1:**
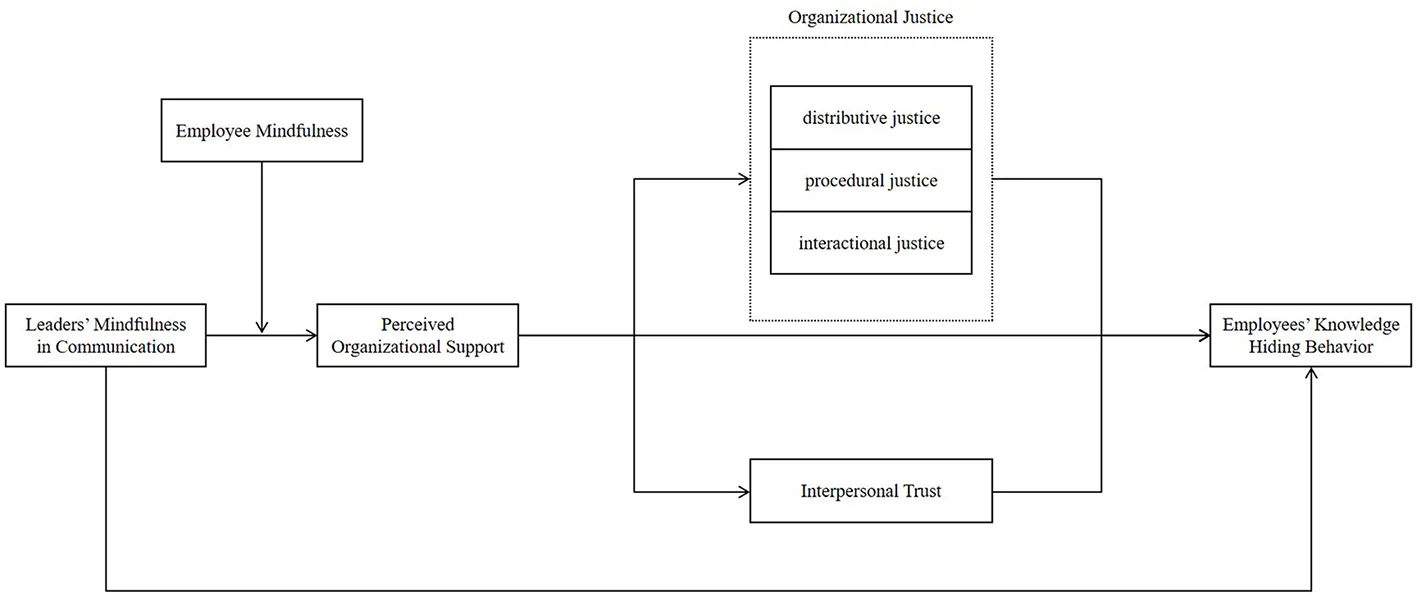
Theoretical model diagram.

We tested this model through two complementary studies. Study 1 used a three-wave survey of 652 employees from Chinese technology firms to examine the proposed moderated model with two serial mediation paths. Study 2 employed a controlled four-week mindfulness training intervention involving 180 leaders and 540 employees to provide additional causal evidence from a field intervention. By combining multi-wave survey data with an intervention design, this research contributes to the literature in three ways. First, it advances research on employees' knowledge hiding behavior by shifting attention from relatively stable leadership styles to a specific and trainable communication behavior. Second, it extends workplace mindfulness research by examining the joint role of leaders' mindfulness in communication and employee mindfulness rather than treating mindfulness as a single-level phenomenon (Good et al., 2016). Third, it integrates social exchange theory and social information processing theory to explain both the relational mechanisms and the individual boundary condition through which mindful communication reduces defensive knowledge behavior. The theoretical model diagram is shown in Figure 1.

## 2 Theory and hypotheses

### 2.1 Social exchange and social information processing

Social exchange theory and social information processing theory provide complementary explanations for how leaders' mindfulness in communication reduces employees' knowledge hiding behavior. Social exchange theory explains the motivational mechanism underlying this relationship. It suggests that workplace relationships are shaped by reciprocal resource exchanges, in which employees evaluate the benefits, costs, and fairness of exchange relationships before forming attitudes and behaviors (Cropanzano and Mitchell, 2005). From this perspective, employees' knowledge hiding behavior can be viewed as a defensive response to perceived imbalance in resource exchange (Serenko and Bontis, 2016). Employees may withhold knowledge when they believe that sharing will weaken their personal advantage, expose their contributions to unfair use, or produce insufficient relational returns (Peng, 2013).

Leaders' mindfulness in communication may interrupt this defensive exchange logic by conveying respect, care, and support (Babič et al., 2019). Such communication can enhance employees' perceived organizational support, which reflects the extent to which employees feel valued and cared for by the organization. This perceived support may then reduce employees' knowledge hiding behavior through two downstream mechanisms. First, it may strengthen organizational justice by increasing employees' expectations that their knowledge contributions will be treated fairly. Second, it may foster interpersonal trust by making knowledge-related interactions appear safer and more reliable. In this way, social exchange theory explains why mindful leader communication can reduce employees' motivation to protect knowledge defensively.

Social information processing theory complements this exchange-based explanation by clarifying how employees interpret leader communication cues. It argues that employees actively attend to and interpret social cues in the workplace before forming attitudes and behavioral responses (Salancik and Pfeffer, 1978). Leaders' mindful communication therefore functions not only as an exchange resource but also as a social cue that signals whether the work environment is supportive, fair, and safe for knowledge sharing. Employee mindfulness shapes this interpretive process. Employees with higher mindfulness are more likely to notice leaders' attentive listening, non-judgmental acceptance, and empathic responding, and to interpret these behaviors as genuine indicators of organizational support. Thus, social information processing theory explains when and for whom leaders' mindfulness in communication is more likely to activate the exchange-based mechanism proposed by social exchange theory.

Taken together, the two theories are complementary rather than parallel. Social information processing theory explains how employees decode leaders' mindful communication as meaningful support-related information, whereas social exchange theory explains why such perceived support subsequently reduces employees' knowledge hiding behavior through enhanced justice perceptions and interpersonal trust. This integrated perspective extends prior research on employees' knowledge hiding behavior by showing that employees' knowledge behavior is shaped not only by the availability of supportive exchange resources but also by employees' capacity to perceive and interpret those resources.

It is also important to distinguish the two mindfulness-related constructs in our model. Leaders' mindfulness in communication refers to an observable and situation-specific leadership behavior, whereas employee mindfulness represents a relatively stable individual disposition (Glomb et al., 2011). Leaders' mindful communication provides high-quality social exchange resources and social information, while employee mindfulness shapes how effectively these resources and information are received and processed. We discuss these relationships in greater detail in the following sections.

### 2.2 Leaders' mindfulness in communication and employees' knowledge hiding behavior

Leaders' mindfulness in communication refers to a communication pattern in which leaders maintain focused, non-judgmental awareness of the present moment and build connections through deep listening and genuine expression during interactions with employees. When leaders demonstrate presence and empathy in communication, they can reduce psychological distance and help employees feel supported and respected (Edmondson, 1999). A supportive communication climate encourages employees to express ideas and share knowledge, whereas a defensive climate may activate knowledge-withholding motives (Cerne et al., 2014). Thus, employees' assessment of relational safety and knowledge-sharing risk is central to their knowledge behavior decisions (Connelly and Zweig, 2015).

Employees' knowledge hiding behavior is defined as an intentional act of concealing or withholding knowledge requested by colleagues (Connelly et al., 2012). According to social exchange theory, employees are less likely to share knowledge when they expect status threats, relational risks, or unfair treatment. Leaders' mindfulness in communication conveys non-judgmental acceptance and empathic concern, signaling that employees' performance and contributions will be noticed and that sharing knowledge is less likely to result in resource loss (Pinck and Sonnentag, 2018). These signals reduce employees' defensive expectations regarding knowledge exposure and weaken their psychological drive for employees' knowledge hiding behavior (Venz and Nesher Shoshan, 2022). Conversely, when employees do not perceive acceptance, empathy, attention, or support in communication with their leader, they may hide knowledge to safeguard their own resources (Offergelt et al., 2019). Based on the above reasoning, we propose the following hypothesis:

Hypothesis 1: Leaders' mindfulness in communication negatively affects employees' knowledge hiding behavior.

### 2.3 The mediating role of perceived organizational support

Perceived organizational support refers to employees' overall perception of the extent to which the organization and its leaders value their contributions and care about their wellbeing. Leaders' mindfulness in communication can enhance perceived organizational support by recognizing employees' needs, providing timely feedback, and respecting their emotional experiences. Because leaders are typically regarded as agents of the organization, employees often interpret leaders' mindful communication as a reflection of organizational intent. When leaders communicate with attention, respect, and care, employees are more likely to infer that the organization itself is supportive and caring. Therefore, leaders' mindfulness in communication may strengthen employees' perceived organizational support. Based on the above reasoning, we propose the following hypothesis:

Hypothesis 2: Leaders' mindfulness in communication positively affects perceived organizational support.

Perceived organizational support can also reduce employees' knowledge hiding behavior. When employees perceive strong organizational support, they develop a felt obligation to reciprocate to the organization (Eisenberger et al., 2001) and are more likely to engage in positive organizational behaviors (Podsakoff et al., 2000). Employees' knowledge hiding behavior is often driven by concerns that sharing knowledge may reduce personal advantage or expose employees to resource loss. Perceived organizational support reduces these concerns by assuring employees that the organization values their contributions and protects their interests (Pereira and Mohiya, 2021). As a result, employees are less likely to treat personal knowledge as a scarce resource to be protected and more likely to view knowledge sharing as a form of social exchange and mutual value creation (Choi et al., 2022). Based on the above reasoning, we propose the following hypothesis:

Hypothesis 3: Perceived organizational support negatively affects employees' knowledge hiding behavior.

Taken together, H2 and H3 suggest that perceived organizational support serves as a psychological bridge linking leaders' mindfulness in communication to employees' knowledge hiding behavior. Supportive signals conveyed through mindful communication strengthen perceived organizational support, which in turn increases employees' sense of security and reciprocation motivation, making them less inclined to hide knowledge. Based on the above reasoning, we propose the following hypothesis:

Hypothesis 4: Perceived organizational support mediates the relationship between leaders' mindfulness in communication and employees' knowledge hiding behavior.

### 2.4 Serial mediation model

Drawing on social exchange theory, we further argue that perceived organizational support is not only a direct psychological bridge but also a critical upstream mechanism that reduces employees' knowledge hiding behavior through more proximal cognitive and affective pathways. Specifically, perceived organizational support may influence employees' knowledge hiding behavior through two parallel serial mediation pathways: organizational justice and interpersonal trust.

First, from a cognitive appraisal perspective, perceived organizational support can translate into concrete judgments about organizational justice (Colquitt, 2001; Huo et al., 2016). When employees feel supported by the organization, they are more likely to believe that their knowledge contributions will be evaluated and treated fairly (Xu et al., 2023). This belief weakens the instrumental motivation to protect knowledge for self-interest. Organizational justice can be understood through distributive justice, procedural justice, and interactional justice (Koay and Lim, 2023). In terms of distributive justice, employees may believe that their knowledge contributions will be fairly reflected in outcomes such as rewards or promotion. In terms of procedural justice, they may trust that organizational procedures and appeal channels can protect their interests if disputes arise from knowledge sharing (Schuh et al., 2019). In terms of interactional justice, the respect and sincerity inherent in mindful communication may reinforce the belief that employees' value is recognized by the organization (Bies and Moag, 1986; Khalid et al., 2018). Thus, perceived organizational support can transform the positive influence of leaders' mindfulness in communication into stronger justice perceptions, thereby weakening the cognitive foundation of employees' knowledge hiding behavior.

Second, from an affective bonding perspective, perceived organizational support may foster interpersonal trust. Research on trust spillover suggests that employees' generalized trust in the organization can extend to trust in other organizational members (Mayer et al., 1995). When employees interpret leaders' mindful communication as evidence that the organization values reciprocity, fairness, and interpersonal safety, they are more likely to believe that colleagues are trustworthy and will not misuse the knowledge they share. This interpersonal trust reduces perceived relational risk and weakens the affective motivation to adopt employees' knowledge hiding behavior as a defensive strategy (Zhang and Takahashi, 2024).

Therefore, perceived organizational support functions as a critical hub in the relationship between leaders' mindfulness in communication and employees' knowledge hiding behavior. It can translate into employees' cognitive judgments about organizational justice and generate affective bonds through interpersonal trust. These two sequential transmission pathways together constitute an important internal mechanism through which leaders' mindfulness in communication curbs employees' knowledge hiding behavior. Because organizational justice comprises distributive justice, procedural justice, and interactional justice, we further examine the three justice dimensions separately. Based on the above reasoning, we propose the following hypotheses:

Hypothesis 5: Leaders' mindfulness in communication negatively affects employees' knowledge hiding behavior through the serial mediation of perceived organizational support and employees' perceptions of organizational justice.
Hypothesis 5a: Leaders' mindfulness in communication negatively affects employees' knowledge hiding behavior through the serial mediation of perceived organizational support and distributive justice.
Hypothesis 5b: Leaders' mindfulness in communication negatively affects employees' knowledge hiding behavior through the serial mediation of perceived organizational support and procedural justice.
Hypothesis 5c: Leaders' mindfulness in communication negatively affects employees' knowledge hiding behavior through the serial mediation of perceived organizational support and interactional justice.
Hypothesis 6: Leaders' mindfulness in communication negatively affects employees' knowledge hiding behavior through the serial mediation of perceived organizational support and interpersonal trust.

### 2.5 The moderating role of employee mindfulness

Employee mindfulness represents an individual trait. Prior research defines it as the ability to maintain awareness and attention to internal and external experiences in the present moment with an open and accepting attitude (Brown and Ryan, 2003). According to social information processing theory, employees rely on social cues in the workplace to form judgments about organizational support and adjust their behavior accordingly. Employee mindfulness shapes this interpretive process by influencing how deeply and accurately employees receive and process social information.

In the context of mindful leader communication, highly mindful employees are more likely to notice leaders' attentive listening, non-judgmental acceptance, and empathic responding. They are also less likely to distort communication cues through defensive reactions, negative filtering, or over-interpretation (Hülsheger et al., 2013). As a result, highly mindful employees are more likely to interpret leaders' mindfulness in communication as an authentic signal of organizational support. Hence, the higher the level of employee mindfulness, the stronger the positive effect of leaders' mindfulness in communication on perceived organizational support. Based on the above reasoning, we propose the following hypothesis:

Hypothesis 7: Employee mindfulness positively moderates the relationship between leaders' mindfulness in communication and perceived organizational support.

## 3 Study 1: The mechanism underlying the effect of leaders' mindfulness in communication on employees' knowledge hiding behavior: the serial mediating roles of perceived organizational support, organizational justice, and interpersonal trust

### 3.1 Participants and procedures

The survey was conducted offline using self-administered paper-and-pencil questionnaires administered at the participating companies. As typical knowledge-intensive enterprises, technology companies have higher demands for knowledge exchange and a more pressing need for the effective flow of knowledge resources. Therefore, Study 1 recruited employees from Chinese technology companies to examine the relationships among leaders' mindfulness in communication, perceived organizational support, organizational justice, interpersonal trust, employees' knowledge hiding behavior, and employee mindfulness.

The research team first contacted senior managers or persons in charge of the participating companies to obtain organizational permission. After permission was granted, each company assigned HR staff to accompany the authors and assist with questionnaire distribution and on-site coordination. The authors were present throughout the data collection process and were responsible for organizing the survey, explaining the research purpose and procedures, answering participants' questions, and collecting the completed questionnaires. HR staff only provided logistical support and did not participate in explaining the study, monitoring participants' responses, or accessing individual questionnaire content. Completed questionnaires were returned directly to the research team in sealed envelopes. This arrangement was adopted to reduce participants' concerns about managerial evaluation and to protect response independence.

Before the survey began, the authors informed participants that participation was voluntary, that the data would be used solely for academic research, and that their responses would remain anonymous and confidential. To match responses across the three waves while protecting anonymity, each participant generated a unique identification code according to a standardized coding rule provided by the research team. This code was used only for matching questionnaires across time points and did not contain directly identifiable personal information.

Given the potential time-lagged effects of leaders' mindfulness in communication, this study adopted a three-wave data collection design with a 4 month interval between adjacent waves. At Time 1, 750 participants reported demographic information and assessed their leaders' mindfulness in communication and their own employee mindfulness. Four months later, at Time 2, participants reported perceived organizational support. Another 4 months later, at Time 3, participants reported organizational justice, interpersonal trust, and employees' knowledge hiding behavior. Questionnaires were excluded if they could not be matched across the three waves, contained substantial missing data, or showed obvious invalid response patterns.

Taking into account employee turnover, job reassignment, and unmatched or invalid responses, 652 valid matched questionnaires were retained, yielding an effective response rate of 86.93%. In the final sample, 406 participants were male and 246 were female, accounting for 62.27% and 37.73%, respectively. The predominant age groups were 26-30 years and 18-25 years, accounting for 31.13% and 28.83%, respectively. Educational attainment was mainly at the bachelor's and master's degree levels, accounting for 49.54% and 24.08%, respectively. The major departments were marketing and R&D, accounting for 44.63% and 26.07%, respectively.

### 3.2 Measures

In this survey, all items—except for demographic variables—were derived from established scales published in prior academic research. In accordance with the requirements of the copyright holders and following academic conventions, all scales were used solely for non-commercial academic research purposes, with full citation of the original sources. The English items were translated into Chinese through a translation and back-translation procedure: two bilingual scholars independently conducted the translation and back-translation, and all discrepancies were resolved through discussion to ensure the clarity, semantic equivalence, and contextual appropriateness of the items. All items were measured on a five-point Likert scale, ranging from 1 (“strongly disagree”) to 5 (“strongly agree”).

#### 3.2.1 Leader mindfulness in communication

We adopted the scale developed by Arendt et al. (2019). This scale consists of nine items, such as “My leader does not become impatient when I speak.” In the present study, the Cronbach's alpha coefficient of this scale was 0.919.

#### 3.2.2 Perceived organizational support

We adopted the scale developed by Lambert (2000). This scale consists of eight items, such as “The organization considers my goals and values.” In the present study, the Cronbach's alpha coefficient of this scale was 0.896.

#### 3.2.3 Organizational justice (including distributive justice, procedural justice, and interactional justice)

We adopted the scale developed by Moorman (1991). This scale comprises three dimensions: distributive justice, procedural justice, and interactional justice. The distributive justice dimension includes five items, such as “Considering your responsibilities, the pay is quite reasonable.” The procedural justice dimension includes seven items, such as “Provides opportunities to challenge decisions.” The interactional justice dimension includes six items, such as “Your supervisor treats you with kindness and consideration.” In the present study, the Cronbach's alpha coefficient of the overall scale was 0.930; the Cronbach's alpha coefficients for the distributive justice, procedural justice, and interactional justice dimensions were 0.866, 0.903, and 0.891, respectively.

#### 3.2.4 Interpersonal trust

We adopted the scale developed by McAllister (1995). This scale consists of ten items, such as “When interacting with colleagues, I find him/her to be trustworthy.” In the present study, the Cronbach's alpha coefficient of this scale was 0.929.

#### 3.2.5 Employees' knowledge hiding behavior

We adopted the scale developed by Connelly et al. (2012). This scale consists of twelve items, such as “I would explain that the information is confidential and only accessible to specific personnel.” In the present study, the Cronbach's alpha coefficient of this scale was 0.942.

#### 3.2.6 Employee mindfulness

We adopted the scale developed by Kimmes et al. (2018). This scale consists of five items, such as “When I talk to my colleagues, we both pay attention to each other's viewpoints.” In the present study, the Cronbach's alpha coefficient of this scale was 0.832.

#### 3.2.7 Control variables

The control variables in this study included age, gender, education level, and department, as these variables may potentially influence employees' knowledge hiding behavior.

### 3.3 Data analysis strategy

We followed a stepwise analytical strategy. First, confirmatory factor analysis was conducted to examine discriminant validity among the core constructs. Second, Harman's single-factor test was used as a diagnostic check for common method bias. Third, descriptive statistics and Pearson correlations were calculated. Fourth, maximum likelihood path analysis was used to test the direct effects among leaders' mindfulness in communication, perceived organizational support, organizational justice, interpersonal trust, and employees' knowledge hiding behavior. Fifth, serial mediation effects were tested using a bootstrap procedure with 5,000 resamples. Finally, hierarchical regression analysis was used to test the moderating effect of employee mindfulness, and simple slope analysis was conducted at one standard deviation above and below the mean of employee mindfulness.

## 4 Results

### 4.1 Confirmatory factor analysis

In the confirmatory factor analysis, the variables included leaders' mindfulness in communication, perceived organizational support, distributive justice, procedural justice, interactional justice, interpersonal trust, employees' knowledge hiding behavior, and employee mindfulness. The fit indices of the eight-factor model (χ^2^ = 2,059.843, df = 1,741, χ^2^/df = 1.183, CFI = 0.986, NFI = 0.919, NNFI = 0.986, TLI = 0.986, IFI = 0.986, RMSEA = 0.017) were superior to those of the alternative models. This indicates that the eight constructs possess good distinctiveness and can be examined in subsequent analyses. The confirmatory factor analysis results are shown in Table 1.

**Table 1:** Confirmatory factor analysis results.

Model	χ^2^	df	χ^2^/df	CFI	NFI	NNFI	TLI	IFI	RMSEA
Eight-factor model (h)	2059.843	1,741	1.183	0.986	0.919	0.986	0.986	0.986	0.017
Seven-factor model (g)	3171.330	1,748	1.814	0.939	0.875	0.936	0.936	0.940	0.035
Six-factor model (f)	3521.087	1,754	2.007	0.925	0.861	0.921	0.921	0.925	0.039
Five-factor model (e)	4627.482	1,759	2.631	0.878	0.817	0.873	0.873	0.878	0.050
Four-factor model (d)	5634.764	1,763	3.196	0.835	0.777	0.829	0.829	0.835	0.058
Three-factor model (c)	6660.876	1,766	3.772	0.791	0.736	0.784	0.784	0.792	0.065
Two-factor model (b)	7809.283	1,768	4.417	0.742	0.691	0.733	0.733	0.743	0.072
One-factor model (a)	9402.112	1,769	5.315	0.674	0.628	0.663	0.663	0.675	0.081

a = Leaders' Mindfulness in Communication + Perceived Organizational Support + Distributive Justice + Procedural Justice + Interactional Justice + Interpersonal Trust + Employees' Knowledge Hiding Behavior + Employee Mindfulness.

b = Leaders' Mindfulness in Communication + Employee Mindfulness + Perceived Organizational Support + Distributive Justice + Procedural Justice + Interactional Justice + Interpersonal Trust; Employees' Knowledge Hiding Behavior.

c = Leaders' Mindfulness in Communication + Employee Mindfulness; Perceived Organizational Support + Distributive Justice + Procedural Justice + Interactional Justice + Interpersonal Trust; Employees' Knowledge Hiding Behavior.

d = Leaders' Mindfulness in Communication + Employee Mindfulness; Perceived Organizational Support; Distributive Justice + Procedural Justice + Interactional Justice + Interpersonal Trust; Employees' Knowledge Hiding Behavior.

e = Leaders' Mindfulness in Communication + Employee Mindfulness; Perceived Organizational Support; Distributive Justice + Procedural Justice + Interactional Justice; Interpersonal Trust; Employees' Knowledge Hiding Behavior.

f = Leaders' Mindfulness in Communication; Perceived Organizational Support; Distributive Justice + Procedural Justice + Interactional Justice; Interpersonal Trust; Employees' Knowledge Hiding Behavior; Employee Mindfulness.

g = Leaders' Mindfulness in Communication + Employee Mindfulness; Perceived Organizational Support; Distributive Justice; Procedural Justice; Interactional Justice; Interpersonal Trust; Employees' Knowledge Hiding Behavior.

h = Leaders' Mindfulness in Communication; Perceived Organizational Support; Distributive Justice; Procedural Justice; Interactional Justice; Interpersonal Trust; Employees' Knowledge Hiding Behavior; Employee Mindfulness.

### 4.2 Common method bias analysis

The data for this study were collected from employees of technology companies. All data were collected at three time points, with a four-month interval between each collection. To further examine the potential influence of common method bias in this study, we conducted Harman's single-factor test. The results indicated that the unrotated first principal component accounted for 38.232% of the variance, which is below the 40% threshold (Podsakoff et al., 2003). These results provide preliminary evidence that common method bias was unlikely to substantially affect the findings.

### 4.3 Descriptive statistical analysis and correlation analysis

Table 2 presents the correlations among the key variables. Leaders' mindfulness in communication was positively correlated with perceived organizational support and negatively correlated with employees' knowledge hiding behavior. Perceived organizational support was positively correlated with distributive justice, procedural justice, interactional justice, and interpersonal trust, and it was negatively correlated with employees' knowledge hiding behavior. Distributive justice, procedural justice, interactional justice, and interpersonal trust were each negatively correlated with employees' knowledge hiding behavior. These results provide preliminary support for the proposed relationships.

**Table 2:** Descriptive statistical analysis and correlation analysis.

Variables	Mean	SD	1	2	3	4	5	6	7	8	9	10	11	12
1. Gender	1.377	0.485	1											
2. Age	2.334	1.159	0.029	1										
3. Education level	2.730	0.928	0.039	−0.309^***^	1									
4. Department	2.094	1.264	−0.033	−0.092^*^	0.424^***^	1								
5. Leaders' mindfulness in communication	3.399	0.980	0.030	0.021	−0.034	−0.055	1							
6. Perceived organizational support	3.423	0.979	0.018	−0.016	0.017	−0.023	0.483^***^	1						
7. Distributive justice	3.397	1.017	0.044	−0.002	−0.023	−0.084^*^	0.504^***^	0.480^***^	1					
8. Procedural justice	3.415	1.009	0.012	0.005	−0.040	−0.056	0.549^***^	0.514^***^	0.524^***^	1				
9. Interactional justice	3.430	1.033	0.004	−0.055	−0.015	−0.051	0.590^***^	0.547^***^	0.548^***^	0.597^***^	1			
10.Interpersonal trust	3.428	0.990	0.038	−0.001	−0.027	−0.061	0.571^***^	0.556^***^	0.548^***^	0.595^***^	0.634^***^	1		
11. Employees' knowledge hiding behavior	2.602	0.990	−0.052	−0.021	−0.005	0.030	−0.560^***^	−0.583^***^	−0.548^***^	−0.581^***^	−0.613^***^	−0.636^***^	1	
12. Employee mindfulness	3.647	0.911	−0.027	−0.031	−0.008	−0.033	0.249^***^	0.514^***^	0.237^***^	0.332^***^	0.324^***^	0.340^***^	−0.316^***^	1

^*^p < 0.05, ^**^p < 0.01, ^***^p < 0.001.

### 4.4 Path model analysis

We used maximum likelihood (ML) estimation to conduct path analysis and model testing, obtaining the path coefficients and significance levels to examine the structural relationships among variables in depth. The results are presented in Table 3.

**Table 3:** Path analysis.

Path	*B*	*SE*	*z*	*p*
Leaders' mindfulness in communication → Perceived organizational support	0.484	0.034	14.105	< 0.001
Leaders' mindfulness in communication → Employees' knowledge hiding behavior	−0.125	0.030	−3.897	< 0.001
Perceived organizational support → Organizational justice	0.612	0.027	22.667	< 0.001
Perceived organizational support → Distributive justice	0.480	0.036	13.987	< 0.001
Perceived organizational support → Procedural justice	0.514	0.035	15.301	< 0.001
Perceived organizational support → Interactional justice	0.546	0.035	16.662	< 0.001
Perceived organizational support → Interpersonal trust	0.556	0.033	17.078	< 0.001
Perceived organizational support → Employees' knowledge hiding behavior	−0.209	0.044	−4.429	< 0.001
Organizational justice → Employees' knowledge hiding behavior	−0.357	0.038	−10.237	< 0.001
Distributive justice → Employees' knowledge hiding behavior	−0.132	0.029	−4.147	< 0.001
Procedural justice → Employees' knowledge hiding behavior	−0.140	0.030	−4.284	< 0.001
Interactional justice → Employees' knowledge hiding behavior	−0.166	0.030	−4.962	< 0.001
Interpersonal trust → Employees' knowledge hiding behavior	−0.233	0.031	−6.925	< 0.001

The results showed that leaders' mindfulness in communication had a significant negative effect on employees' knowledge hiding behavior (*B* = −0.125, SE = 0.030, *z* = −3.897, *p* < 0.001), supporting Hypothesis 1. Leaders' mindfulness in communication had a significant positive effect on perceived organizational support (*B* = 0.484, SE = 0.034, *z* = 14.105, *p* < 0.001), supporting Hypothesis 2. Perceived organizational support had a significant negative effect on employees' knowledge hiding behavior (*B* = −0.209, SE = 0.044, *z* = −4.429, *p* < 0.001), supporting Hypothesis 3. These results provide preliminary support for the component paths underlying Hypothesis 4. In addition, perceived organizational support had significant positive effects on overall organizational justice (*B* = 0.612, SE = 0.027, *z* = 22.667, *p* < 0.001), distributive justice (*B* = 0.480, SE = 0.036, *z* = 13.987, *p* < 0.001), procedural justice (*B* = 0.514, SE = 0.035, *z* = 15.301, *p* < 0.001), interactional justice (*B* = 0.546, SE = 0.035, *z* = 16.662, *p* < 0.001), and interpersonal trust (*B* = 0.556, SE = 0.033, *z* = 17.078, *p* < 0.001). Meanwhile, overall organizational justice (*B* = −0.357, SE = 0.038, *z* = −10.237, *p* < 0.001), distributive justice (*B* = −0.132, SE = 0.029, *z* = −4.147, *p* < 0.001), procedural justice (*B* = −0.140, SE = 0.030, *z* = −4.284, *p* < 0.001), interactional justice (*B* = −0.166, SE = 0.030, *z* = −4.962, *p* < 0.001), and interpersonal trust (*B* = −0.233, SE = 0.031, *z* = −6.925, *p* < 0.001) had significant negative effects on employees' knowledge hiding behavior. These results provide preliminary support for the component paths underlying Hypotheses 5, 5a, 5b, 5c, and 6.

### 4.5 Serial mediation analysis

We used a bootstrap procedure with 5,000 resamples to test the indirect effects of leaders' mindfulness in communication on employees' knowledge hiding behavior. The results are reported in Table 4. Because none of the 95% confidence intervals included zero, all proposed indirect effects were statistically significant.

**Table 4:** Serial mediation analysis.

Path	*B*	*SE*	*t*	*p*	95% CI
A: Leaders' mindfulness in communication → Perceived organizational support → Employees' knowledge hiding behavior	−0.199	0.033	−6.111	< 0.001	[−0.267, −0.141]
B: Leaders' mindfulness in communication → Perceived organizational support → Organizational justice → Employees' knowledge hiding behavior	−0.084	0.015	−5.441	< 0.001	[−0.115, −0.056]
C: Leaders' mindfulness in communication → Perceived organizational support → Distributive justice → Employees' knowledge hiding behavior	−0.038	0.009	−4.119	< 0.001	[−0.058, −0.022]
D: Leaders' mindfulness in communication → Perceived organizational support → Procedural justice → Employees' knowledge hiding behavior	−0.044	0.011	−4.201	< 0.001	[−0.067, −0.025]
E: Leaders' mindfulness in communication → Perceived organizational support → Interactional justice → Employees' knowledge hiding behavior	−0.052	0.012	−4.464	< 0.001	[−0.077, −0.032]
F: Leaders' mindfulness in communication → Perceived organizational support → Interpersonal trust → Employees' knowledge hiding behavior	−0.063	0.013	−4.835	< 0.001	[−0.091, −0.040]

First, Path A examined the simple mediating effect of perceived organizational support in the relationship between leaders' mindfulness in communication and employees' knowledge hiding behavior. The indirect effect was significant and negative, B = −0.199, SE = 0.033, *t* = –6.111, *p* < 0.001, 95% CI [−0.267, −0.141], supporting Hypothesis 4. This finding indicates that leaders' mindful communication reduces employees' knowledge hiding partly by increasing employees' perception that the organization values and supports them.

Second, Path B tested the serial mediation effect through perceived organizational support and overall organizational justice. This indirect effect was also significant and negative, *B* = −0.084, SE = 0.015, *t* = −5.441, *p* < 0.001, 95% CI [−0.115, −0.056], supporting Hypothesis 5. This result suggests that leaders' mindful communication enhances perceived organizational support, which further strengthens employees' overall justice perceptions and thereby reduces knowledge hiding.

Third, Paths C, D, and E further decomposed organizational justice into distributive justice, procedural justice, and interactional justice. The indirect effects were significant for distributive justice, *B* = −0.038, SE = 0.009, *t* = −4.119, *p* < 0.001, 95% CI [−0.058, −0.022], procedural justice, *B* = −0.044, SE = 0.011, *t* = −4.201, *p* < 0.001, 95% CI [−0.067, −0.025], and interactional justice, *B* = −0.052, SE = 0.012, *t* = −4.464, *p* < 0.001, 95% CI [−0.077, −0.032]. These findings support Hypotheses 5a, 5b, and 5c, respectively. They indicate that perceived organizational support reduces knowledge hiding not only by improving employees' general fairness perceptions, but also by strengthening their beliefs that outcomes, procedures, and interpersonal treatment within the organization are fair.

Finally, Path F examined the serial mediation effect through perceived organizational support and interpersonal trust. The indirect effect was significant and negative, *B* = −0.063, SE = 0.013, *t* = −4.835, *p* < 0.001, 95% CI [−0.091, −0.040], supporting Hypothesis 6. This result shows that leaders' mindful communication can increase perceived organizational support, foster interpersonal trust, and subsequently reduce employees' tendency to hide knowledge.

Overall, these results provide consistent evidence for the proposed mediation model. Leaders' mindfulness in communication reduces employees' knowledge hiding behavior not only directly through perceived organizational support, but also indirectly through two downstream mechanisms: enhanced organizational justice and strengthened interpersonal trust.

### 4.6 Moderation analysis

We adopted the moderation testing procedure proposed by Wen et al. (2005) and Preacher and Hayes (2008) to examine whether employee mindfulness moderated the relationship between leaders' mindfulness in communication and perceived organizational support. The results are presented in Table 5. Before creating the interaction term, leaders' mindfulness in communication and employee mindfulness were mean-centered to reduce potential multicollinearity.

**Table 5:** Moderation analysis.

Variables	Dependent variable: perceived organizational support		
	Model 1	Model 2	Model 3
**Control variables**			
Gender	0.005 (SE = 0.070, *t* = 0.069)	0.034 (SE = 0.062, *t* = 0.553)	0.040 (SE = 0.062, *t* = 0.655)
Age	−0.015 (SE = 0.031, *t* = −0.476)	−0.001 (SE = 0.027, *t* = −0.047)	-0.002 (SE = 0.027, *t* = −0.079)
Education level	0.035 (SE = 0.042, *t* = 0.824)	0.035 (SE = 0.037, *t* = 0.929)	0.034 (SE = 0.037, *t* = 0.914)
Department	−0.009 (SE = 0.030, *t* = −0.304)	−0.001 (SE = 0.026, *t* = −0.043)	-0.003 (SE = 0.026, *t* = −0.124)
Independent variable			
Leaders' mindfulness in communication	0.484^***^ (SE = 0.034, *t* = 14.044)	0.379^***^ (SE = 0.032, *t* = 12.032)	0.375^***^ (SE = 0.031, *t* = 11.939)
Moderator			
Employee mindfulness		0.452^***^ (SE = 0.034, *t* = 13.345)	0.492^***^ (SE = 0.037, *t* = 13.298)
Interactive term			
Leaders' mindfulness in communication × Employee mindfulness			0.072^**^ (SE = 0.027, *t* = 2.628)
*N*	652	652	652
*R^2^*	0.235	0.401	0.407
*ΔR^2^*	0.235	0.165	0.006
*F*	*F* (5, 646) = 39.743, *p* < 0.001	*F*(6, 645) = 71.880, *p* < 0.001	*F*(7, 644) = 63.162, *p* < 0.001

^*^ *p* < 0.05, ^**^ *p* < 0.01, ^***^ *p* < 0.001.

Hierarchical regression analysis was conducted in three steps. In Model 1, the control variables and leaders' mindfulness in communication were entered. In Model 2, employee mindfulness was added as the moderator. In Model 3, the interaction term between leaders' mindfulness in communication and employee mindfulness was entered. As shown in Model 3, the interaction term was positively and significantly associated with perceived organizational support, *B* = 0.072, SE = 0.027, *t* = 2.628, *p* < 0.01. This result indicates that employee mindfulness strengthened the positive relationship between leaders' mindfulness in communication and perceived organizational support, thereby supporting Hypothesis 7.

Specifically, when employees had higher levels of mindfulness, they were more likely to notice and accurately interpret leaders' attentive listening, non-judgmental acceptance, and empathic responses as signals of organizational support. Therefore, leaders' mindfulness in communication had a stronger positive effect on perceived organizational support among highly mindful employees. In contrast, when employees had lower levels of mindfulness, they may have been less attentive to such supportive communication cues or less able to process them in an open and non-defensive manner. As a result, the positive effect of leaders' mindfulness in communication on perceived organizational support was weaker among employees with lower mindfulness.

These findings are consistent with social information processing theory, which suggests that employees' interpretation of workplace social cues depends on their attentional and cognitive processing characteristics. Employee mindfulness therefore functions as an important boundary condition that determines when leaders' mindfulness in communication is more likely to translate into stronger perceived organizational support.

### 4.7 Simple slope analysis

**Figure 2:**
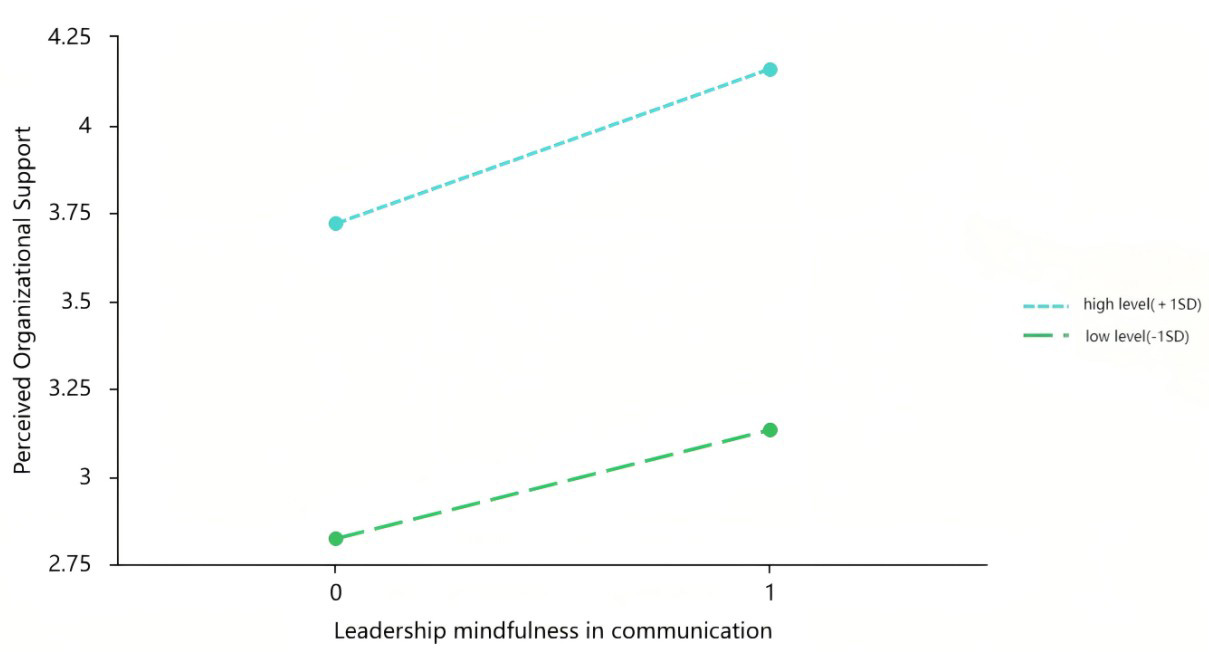
Simple slope analysis.

We conducted a simple slope analysis to examine the effect of leaders' mindfulness in communication on perceived organizational support at high and low levels of employee mindfulness (*M* ± 1 SD). Figure 2 illustrates the relationship between the independent variable and the mediator at different levels of employee mindfulness.

As shown in Table 6, the simple slopes were positive at the mean level of employee mindfulness [*B* = 0.375, SE = 0.031, 95% CI (0.313, 0.437)], high level of employee mindfulness [+1 SD; *B* = 0.440, SE = 0.039, 95% CI (0.364, 0.517)], and low level of employee mindfulness [−1 SD; *B* = 0.309, SE = 0.041, 95% CI (0.229, 0.390)]. Because none of the 95% confidence intervals included zero, these results confirm that employee mindfulness positively moderates the relationship between leaders' mindfulness in communication and perceived organizational support.

**Table 6:** Simple slope analysis.

Moderating effect	Level	*B*	*SE*	95% CI
H7	Mean	0.375	0.031	[0.313, 0.437]
	High level (+1 SD)	0.440	0.039	[0.364, 0.517]
	Low level(−1 SD)	0.309	0.041	[0.229, 0.390]

## 5 Study 2: The mitigating effect of leaders' mindfulness in communication and employee mindfulness trait on employees' knowledge hiding behavior: evidence from a mindfulness training intervention

Prior research indicates that mindfulness can be acquired through training, and meta-analytic evidence shows that abbreviated mindfulness programs are as effective as the traditional 8-week format (Carmody and Baer, 2009), with 4 week programs producing significant improvements that persist for at least 3 months (Rana et al., 2022). Accordingly, this study implemented a 4-week mindfulness training program to further explore whether leaders' and employees' participation in specialized mindfulness training can more effectively reduce employees' knowledge hiding behavior.

### 5.1 Participants

This study employed a 3 (group: control group, experimental group 1, experimental group 2) × 2 (measurement time: pretest, posttest) mixed design, with group as a between-subjects factor and measurement time as a within-subjects factor. An a priori power analysis conducted with G^*^Power (Faul et al., 2007) indicated that a total of 159 participants would be required to detect a medium effect (*f* = 0.25) in a three-group comparison at α = 0.05 with a statistical power of 0.80; our sample size substantially exceeded this threshold. In collaboration with technology companies, we recruited 180 in-service leaders and 540 in-service employees from Jiangsu Province, China. Each leader participated together with three of his or her direct subordinates, forming 180 leader-team units. To prevent within-team contamination, randomization was conducted at the leader-team level: an independent researcher who was not involved in recruitment, training, or data collection used a computer-generated random number sequence, stratified by company, to assign the 180 units evenly to the three groups (60 leaders and 180 employees per group). The final valid sample consisted of 720 participants, of whom 41.81% were female, with a mean age of 33 years. 100758316675

### 5.2 Procedure

The study consisted of three phases: pretest, training, and post-training assessment. All participants completed the pretest and the relevant post-training assessments, and the scales used in these measurements were identical to those used in Study 1. The specific experimental procedure is described below.

First, with the support of the technology companies, we screened leaders and employees who had not previously received any mindfulness training and obtained informed consent from all participants. After successful screening and with participants' consent, we measured leaders' mindfulness in communication among the 180 leaders and employee mindfulness among the 540 employees.

Second, rigorous organization and intervention procedures were implemented during the 4-week training phase. In the control group, neither leaders nor employees received mindfulness training. In experimental group 1, leaders received mindfulness in communication training, whereas employees did not receive mindfulness training. In experimental group 2, leaders received mindfulness in communication training, and employees received mindfulness training.

Each training program comprised 20 sessions delivered 5 days per week (Monday to Friday) over the 4-week training phase, with each session lasting 90 min and each trained participant receiving 30 contact h. Each session followed a standardized three-part structure: an instructor-led explanation of the day's theme and practice essentials (approximately 20 min), guided group practice led by the instructor (approximately 50 min), and a question-and-answer segment in which the instructor addressed participants' questions and corrected practice errors (approximately 20 min). For leaders, the training program focused primarily on mindfulness in communication and progressed from foundational practices to applied workplace exercises: Week 1 cultivated basic attentional and awareness skills (e.g., mindful breathing, body scan, thought observation); Week 2 trained attentive listening (e.g., paired listening exercises, awareness of mind-wandering and bodily signals during listening); Week 3 trained non-judgmental acceptance and empathic responding (e.g., recognizing judgmental impulses, loving-kindness meditation, empathic expression); and Week 4 integrated these skills into workplace scenarios through role-playing exercises (e.g., delivering performance feedback, responding to subordinates' requests for help, and handling disagreements mindfully). For employees, the training program covered mindfulness in communication, mindful breathing, loving-kindness meditation, and mindful stretching, following a parallel 4 week progression: Week 1 covered foundational practices (mindful breathing, body scan, and mindful stretching); Week 2 deepened awareness skills (thought observation, emotion labeling, and loving-kindness meditation); Week 3 focused on mindful communication (attentive listening, non-judgmental acceptance, and mindful expression in simulated colleague interactions); and Week 4 integrated these practices into workplace situations (e.g., brief breathing exercises under work pressure and mindful responses in knowledge-collaboration scenarios).

To ensure intervention fidelity, all training sessions were delivered by the same professionally certified instructor specializing in mindfulness training (Lange and Rowold, 2019), following a standardized training manual that fixed the content, sequence, and duration of each session. One author observed each session and completed a intervention quality checklist to verify that the protocol was implemented as designed, and attendance was recorded at every session to ensure consistency of the training dose across participants. The intervention quality checklist was completed for every training session throughout the 4 week intervention. The checklist included several items designed to verify whether the training protocol was implemented consistently: whether the instructor covered all planned core components specified in the standardized manual; whether the session followed the prescribed sequence of activities; whether the approximate duration of each segment was maintained, including the theme explanation, guided practice, and question-and-answer/correction segment; whether the prescribed practice materials and exercises were used; whether participants were guided to complete the required in-session practices; and whether attendance and participant engagement were recorded. After each session, the observing author reviewed the checklist and noted whether any deviations from the standardized protocol had occurred. Across all scheduled training sessions, the instructor covered the planned core content, followed the intended session structure, and maintained the scheduled training dose. No major deviations from the training protocol were observed.

Several measures were taken to control for contamination. Training sessions for leaders and employees, as well as for different groups, were conducted at separate times and venues, and trained participants were explicitly instructed not to discuss or share any training content with colleagues outside their own group. All non-trained participants—that is, all participants in the control group and all employee participants in experimental group 1—completed a brief standardized daily online report indicating whether they had been exposed to any mindfulness-related content through any channel. Two of the authors monitored these daily self-reports throughout the study and followed up on any irregular or missing reports.

Finally, after the training, we measured leaders' mindfulness in communication and employees' mindfulness in all three groups. Because the effects of training may take time to manifest in the workplace and may involve a time lag, we measured all employees' knowledge hiding behavior 14 days after the training ended, which also allowed us to examine whether the training effects persisted beyond the immediate post-training period. The authors responsible for monitoring the non-trained participants reported that these participants had not been exposed to any mindfulness training content during the experiment. Specifically, across the 4-week training period, non-trained participants (all 240 participants in the control group and all 180 employee participants in experimental group 1, totaling 420 individuals) submitted 8,400 daily reports (5 days/week × 4 weeks × 420 individuals), with a compliance rate of 100%. Zero reports indicated exposure to any mindfulness-related content.

### 5.3 Experimental results

First, this study employed analysis of variance (ANOVA) to assess pretest levels of leaders' mindfulness in communication, perceived organizational support, employees' knowledge hiding behavior, and employee mindfulness across the control group, experimental group 1, and experimental group 2. As shown in Table 7, the initial levels of all variables were comparable across the three groups. Specifically, all ANOVA results were non-significant, with *p* > 0.05. In addition, η^2^ < 0.01 and Cohen's *f* < 0.10, indicating very small between-group differences. These findings demonstrate that the pretest data did not reveal significant differences among the three groups, confirming that the allocation of participants across groups was appropriate and suitable for further experimental investigation.

**Table 7:** ANOVA results for pretest measures across groups.

Variable	Mean ±SD			SE			*F*	*p*	SSB	SST	η^2^	Cohen's f	95% CI		
	Control Group Pretest (*N* = 180)	Experimental Group 1 Pretest (*N* = 180)	Experimental Group 2 Pretest (*N* = 180)	Control Group Pretest (*N* = 180)	Experimental Group 1 Pretest (*N* = 180)	Experimental Group 2 Pretest (*N* = 180)							Control Group Pretest (*N* = 180)	Experimental Group 1 Pretest (*N* = 180)	Experimental Group 2 Pretest (*N* = 180)
Leaders' mindfulness in communication	3.10 ± 0.92	3.10 ± 0.99	3.17 ± 1.02	0.069	0.074	0.076	0.295	0.745	0.564	513.148	0.001	0.033	[2.964, 3.236]	[2.954, 3.246]	[3.020, 3.320]
Perceived organizational support	3.04 ± 0.97	3.07 ± 1.04	3.18 ± 1.07	0.072	0.078	0.080	0.952	0.386	2.015	570.199	0.004	0.060	[2.898, 3.182]	[2.916, 3.224]	[3.022, 3.338]
Employees' knowledge hiding behavior	2.96 ± 0.96	2.98 ± 1.01	2.83 ± 1.01	0.072	0.075	0.075	1.142	0.320	2.259	533.293	0.004	0.065	[2.818, 3.102]	[2.832, 3.128]	[2.682, 2.978]
Employee mindfulness	3.32 ± 0.89	3.35 ± 0.92	3.47 ± 0.85	0.066	0.069	0.063	1.405	0.246	2.197	421.895	0.005	0.072	[3.190, 3.450]	[3.214, 3.486]	[3.346, 3.594]

Second, this study employed independent-samples *t*-tests to examine the effectiveness of leaders' mindfulness in communication training and employees' mindfulness training, thereby ensuring that the premises of the experiment were meaningful. Table 8 presents means, standard deviations, standard errors, *t*-values, *p-*values, and 95% confidence intervals for leaders' mindfulness in communication, perceived organizational support, employees' knowledge hiding behavior, and employee mindfulness at pretest and posttest.

**Table 8:** Pretest - Posttest analysis of mindfulness training effectiveness.

Variable	Group	Measurement Time	*N*	Mean	SD	SE	95% CI	*t*	*p*	Cohen's *d*
Leaders' mindfulness in communication	Control Group	Pretest	180	3.10	0.92	0.101	[−0.197, 0.200]	0.012	0.990	0.001
	Experimental Group 1	Pretest	180	3.10	0.99					
	Control Group	Pretest	180	3.10	0.92	0.102	[−0.269, 0.133]	−0.664	0.507	0.070
	Experimental Group 2	Pretest	180	3.17	1.02					
	Experimental Group 1	Pretest	180	3.10	0.99	0.106	[−0.277, 0.139]	−0.654	0.514	0.069
	Experimental Group 2	Pretest	180	3.17	1.02					
	Control Group	Pretest	180	3.10	0.92	0.099	[−0.192, 0.198]	0.031	0.975	0.003
	Control Group	Posttest	180	3.10	0.96					
	Experimental Group 1	Pretest	180	3.10	0.99	0.101	[−0.591, −0.193]	−3.873	< 0.001	0.408
	Experimental Group 1	Posttest	180	3.49	0.93					
	Experimental Group 2	Pretest	180	3.17	1.02	0.100	[−0.729, −0.337]	−5.354	< 0.001	0.564
	Experimental Group 2	Posttest	180	3.70	0.87					
	Control Group	Posttest	180	3.10	0.96	0.100	[−0.590, −0.198]	−3.956	< 0.001	0.417
	Experimental Group 1	Posttest	180	3.49	0.93					
	Control Group	Posttest	180	3.10	0.96	0.100	[−0.794, −0.415]	−6.267	< 0.001	0.661
	Experimental Group 2	Posttest	180	3.70	0.87					
	Experimental Group 1	Posttest	180	3.49	0.93	0.095	[−0.397, −0.024]	−2.221	< 0.050	0.234
	Experimental Group 2	Posttest	180	3.70	0.87					
Perceived organizational support	Control Group	Pretest	180	3.04	0.97	0.106	[−0.240, 0.178]	−0.294	0.769	0.031
	Experimental Group 1	Pretest	180	3.07	1.04					
	Control Group	Pretest	180	3.04	0.97	0.108	[−0.354, 0.069]	−1.323	0.187	0.139
	Experimental Group 2	Pretest	180	3.18	1.07					
	Experimental Group 1	Pretest	180	3.07	1.04	0.111	[−0.330, 0.108]	−0.997	0.319	0.105
	Experimental Group 2	Pretest	180	3.18	1.07					
	Control Group	Pretest	180	3.04	0.97	0.100	[−0.311, 0.081]	−1.156	0.249	0.122
	Control Group	Posttest	180	3.15	0.92					
	Experimental Group 1	Pretest	180	3.07	1.04	0.106	[−0.515, −0.099]	−2.902	< 0.010	0.306
	Experimental Group 1	Posttest	180	3.37	0.96					
	Experimental Group 2	Pretest	180	3.18	1.07	0.104	[−0.701, −0.294]	−4.803	< 0.001	0.506
	Experimental Group 2	Posttest	180	3.68	0.89					
	Control Group	Posttest	180	3.15	0.92	0.099	[−0.418, −0.028]	−2.245	< 0.050	0.237
	Experimental Group 1	Posttest	180	3.37	0.96					
	Control Group	Posttest	180	3.15	0.92	0.095	[−0.712, −0.337]	−5.499	< 0.001	0.580
	Experimental Group 2	Posttest	180	3.68	0.89					
	Experimental Group 1	Posttest	180	3.37	0.96	0.098	[−0.493, −0.110]	−3.094	< 0.010	0.326
	Experimental Group 2	Posttest	180	3.68	0.89					
Employees' knowledge hiding behavior	Control Group	Pretest	180	2.96	0.96	0.104	[−0.230, 0.179]	−0.244	0.807	0.026
	Experimental Group 1	Pretest	180	2.98	1.01					
	Control Group	Pretest	180	2.96	0.96	0.104	[−0.081, 0.327]	1.182	0.238	0.125
	Experimental Group 2	Pretest	180	2.83	1.01					
	Experimental Group 1	Pretest	180	2.98	1.01	0.107	[−0.061, 0.358]	1.391	0.165	0.147
	Experimental Group 2	Pretest	180	2.83	1.01					
	Control Group	Pretest	180	2.96	0.96	0.102	[−0.225, 0.176]	−0.241	0.810	0.025
	Control Group	Posttest	180	2.98	0.97					
	Experimental Group 1	Pretest	180	2.98	1.01	0.102	[0.168, 0.571]	3.613	< 0.001	0.381
	Experimental Group 1	Posttest	180	2.61	0.92					
	Experimental Group 2	Pretest	180	2.83	1.01	0.099	[0.303, 0.693]	5.022	< 0.001	0.529
	Experimental Group 2	Posttest	180	2.34	0.87					
	Control Group	Posttest	180	2.98	0.97	0.100	[0.172, 0.565]	3.691	< 0.001	0.389
	Experimental Group 1	Posttest	180	2.61	0.92					
	Control Group	Posttest	180	2.98	0.97	0.097	[0.454, 0.836]	6.647	< 0.001	0.701
	Experimental Group 2	Posttest	180	2.34	0.87					
	Experimental Group 1	Posttest	180	2.61	0.92	0.094	[0.091, 0.462]	2.924	< 0.010	0.308
	Experimental Group 2	Posttest	180	2.34	0.87					
Employee mindfulness	Control Group	Pretest	180	3.32	0.89	0.095	[−0.217, 0.157]	−0.316	0.752	0.035
	Experimental Group 1	Pretest	180	3.35	0.92					
	Control Group	Pretest	180	3.32	0.89	0.092	[−0.328, 0.032]	−1.616	0.107	0.170
	Experimental Group 2	Pretest	180	3.47	0.85					
	Experimental Group 1	Pretest	180	3.35	0.92	0.093	[−0.301, 0.065]	−1.265	0.207	0.133
	Experimental Group 2	Pretest	180	3.47	0.85					
	Control Group	Pretest	180	3.32	0.89	0.098	[−0.208, 0.177]	−0.159	0.874	0.017
	Control Group	Posttest	180	3.34	0.97					
	Experimental Group 1	Pretest	180	3.35	0.92	0.093	[−0.366, −0.001]	−1.975	< 0.050	0.208
	Experimental Group 1	Posttest	180	3.54	0.85					
	Experimental Group 2	Pretest	180	3.47	0.85	0.085	[−0.518, −0.186]	−4.166	< 0.001	0.439
	Experimental Group 2	Posttest	180	3.82	0.75					
	Control Group	Posttest	180	3.34	0.97	0.096	[−0.388, −0.010]	−2.066	< 0.050	0.218
	Experimental Group 1	Posttest	180	3.54	0.85					
	Control Group	Posttest	180	3.34	0.97	0.091	[−0.664, −0.305]	−5.298	< 0.001	0.558
	Experimental Group 2	Posttest	180	3.82	0.75					
	Experimental Group 1	Posttest	180	3.54	0.85	0.085	[−0.452, −0.119]	−3.365	< 0.010	0.355
	Experimental Group 2	Posttest	180	3.82	0.75					

With regard to leaders' mindfulness in communication, there were no significant differences between the control group pretest and experimental group 1 pretest (*p* = 0.990, 95% CI included zero), between the control group pretest and experimental group 2 pretest (*p* = 0.507, 95% CI included zero), or between experimental group 1 pretest and experimental group 2 pretest (*p* = 0.514, 95% CI included zero), further confirming that the group allocation was reasonable. The control group showed no significant difference between pretest and posttest (*p* = 0.975, 95% CI included zero), whereas experimental group 1 showed a significant difference between pretest and posttest (*p* < 0.01, 95% CI excluded zero), as did experimental group 2 (*p* < 0.01, 95% CI excluded zero). The mean score for leaders' mindfulness in communication increased by 0.39 from pretest to posttest in experimental group 1 and by 0.53 in experimental group 2, confirming that the mindfulness in communication training for leaders was effective in both experimental groups.

For perceived organizational support, there were no significant differences between the control group pretest and experimental group 1 pretest (*p* = 0.769, 95% CI included zero), between the control group pretest and experimental group 2 pretest (*p* = 0.187, 95% CI included zero), or between experimental group 1 pretest and experimental group 2 pretest (*p* = 0.319, 95% CI included zero), again confirming that the group allocation was appropriate. The control group exhibited no significant difference between pretest and posttest (*p* = 0.249, 95% CI included zero), whereas experimental group 1 showed a significant difference between pretest and posttest (*p* < 0.01, 95% CI excluded zero), as did experimental group 2 (*p* < 0.01, 95% CI excluded zero). Significant differences emerged between control group posttest and experimental group 1 posttest (*p* < 0.05, 95% CI excluded zero), between control group posttest and experimental group 2 posttest (*p* < 0.01, 95% CI excluded zero), and between experimental group 1 posttest and experimental group 2 posttest (*p* < 0.05, 95% CI excluded zero). The mean score for perceived organizational support increased by 0.30 from pretest to posttest in experimental group 1 and by 0.50 in experimental group 2. These findings provide preliminary evidence that the combined implementation of leaders' mindfulness in communication training and employees' mindfulness training was superior to leaders' mindfulness in communication training alone in enhancing perceived organizational support, and that leaders' mindfulness in communication training alone was more effective than no mindfulness training.

For employees' knowledge hiding behavior, there were no significant differences between the control group pretest and experimental group 1 pretest (*p* = 0.807, 95% CI included zero), between the control group pretest and experimental group 2 pretest (*p* = 0.238, 95% CI included zero), or between experimental group 1 pretest and experimental group 2 pretest (*p* = 0.165, 95% CI included zero), once again confirming that the group allocation was reasonable. The control group displayed no significant difference between pretest and posttest (*p* = 0.810, 95% CI included zero), whereas experimental group 1 exhibited a significant difference between pretest and posttest (*p* < 0.01, 95% CI excluded zero), as did experimental group 2 (*p* < 0.01, 95% CI excluded zero). Significant differences were found between control group posttest and experimental group 1 posttest (*p* < 0.05, 95% CI excluded zero), between control group posttest and experimental group 2 posttest (*p* < 0.01, 95% CI excluded zero), and between experimental group 1 posttest and experimental group 2 posttest (*p* < 0.05, 95% CI excluded zero). The mean score for employees' knowledge hiding behavior decreased by 0.37 from pretest to posttest in experimental group 1 and by 0.49 in experimental group 2. These findings provide preliminary evidence that the combined implementation of leaders' mindfulness in communication training and employees' mindfulness training was superior to leaders' mindfulness in communication training alone in reducing employees' knowledge hiding behavior, and that leaders' mindfulness in communication training alone was more effective than no mindfulness training.

For employee mindfulness, there were no significant differences between the control group pretest and experimental group 1 pretest (*p* = 0.752, 95% CI included zero), between the control group pretest and experimental group 2 pretest (*p* = 0.107, 95% CI included zero), or between experimental group 1 pretest and experimental group 2 pretest (*p* = 0.207, 95% CI included zero), again confirming that the group allocation was appropriate. The control group showed no significant difference between pretest and posttest (*p* = 0.874, 95% CI included zero), whereas experimental group 1 exhibited a marginal significant difference between pretest and posttest (*p* = 0.049, 95% CI excluded zero), and experimental group 2 showed a significant difference between pretest and posttest (*p* < 0.01, 95% CI excluded zero). Notably, employee mindfulness in experimental group 1 showed a marginally significant increase from pretest to posttest (*p* = 0.049, 95% CI excluded zero), despite the fact that employees in this group did not receive direct mindfulness training. This finding may reflect an indirect spillover effect of leaders' mindfulness in communication: when leaders exhibit more attentive listening and non-judgmental communication styles, employees may experience modest increases in mindfulness through observational learning or relationship improvement. However, this effect was substantially weaker than that observed in experimental group 2, where employees received direct training (employee mindfulness increased by 0.35 in experimental group 2, *p* < 0.01; the increase in experimental group 1 was smaller and only marginally significant), indicating that direct training was significantly more effective than indirect influence in enhancing employee mindfulness. This finding is consistent with the core hypothesis of this study—that dual intervention produces the strongest effect—while also revealing the potential radiating influence of leaders' behavioral change on employees.

Finally, this study conducted moderated mediation regression analyses using posttest data from the control group, experimental group 1, and experimental group 2. The regression results are presented in Table 9. In Models 1, 3, and 5, posttest data from all three groups supported the inhibitory effect of leaders' mindfulness in communication on employees' knowledge hiding behavior, thereby reconfirming H1. Posttest data from all three groups also supported the inhibitory effect of perceived organizational support on employees' knowledge hiding behavior, thereby reconfirming H3. In Models 2, 4, and 6, posttest data from all three groups supported the positive effect of leaders' mindfulness in communication on perceived organizational support, thereby reconfirming H2. Based on these findings, posttest data from all three groups supported the mediating role of perceived organizational support in the relationship between leaders' mindfulness in communication and employees' knowledge hiding behavior, thereby reconfirming H4. Furthermore, in Models 2, 4, and 6, posttest data from all three groups supported the positive moderating effect of employee mindfulness on the relationship between leaders' mindfulness in communication and perceived organizational support, thereby reconfirming H7.

**Table 9:** Moderated mediation regression model.

Variable	Control Group Posttest		Experimental Group 1 Posttest		Experimental Group 2 Posttest	
	Model 1: employees' knowledge hiding behavior	Model 2: Perceived organizational support	Model 3: Employees'knowledge hiding behavior	Model 4: Perceived organizational support	Model 5: Employees'knowledge hiding behavior	Model 6: Perceived organizational support
Leaders' mindfulness in communication	−0.214^**^ (SE = 0.078, *t* = −2.725)	0.358^***^ (SE = 0.054, *t* = 6.582)	−0.323^***^ (SE = 0.075, *t* = −4.297)	0.384^***^ (SE = 0.060, *t* = 6.395)	−0.362^***^ (SE = 0.075, *t* = −4.824)	0.318^***^ (SE = 0.063, *t* = 5.038)
Employee mindfulness		0.510^***^ (SE = 0.060, *t* = 8.487)		0.486^***^ (SE = 0.065, *t* = 7.460)		0.451^***^ (SE = 0.076, *t* = 5.925)
Leaders' mindfulness in communication × Employee mindfulness		0.111^*^ (SE = 0.052, t = 2.140)		0.151^*^ (SE = 0.063, t = 2.404)		0.236^**^ (SE = 0.083, *t* = 2.842)
Perceived organizational support	−0.323^***^ (SE = 0.082, *t* = −3.957)		−0.215^**^ (SE = 0.073, *t* = − 2.957)		−0.301^***^ (SE = 0.074, *t* = − 4.091)	
*N*	180	180	180	180	180	180
*R* ^2^	0.204	0.478	0.226	0.442	0.358	0.548
Δ*R*^2^	0.19	0.466	0.213	0.429	0.347	0.537
*F*	*F*_(2, 177)_ = 22.687, *p* < 0.001	*F* (_3, 176_) = 53.633, *p* < 0.001	*F*_(2, 177)_ = 25.824, *p* < 0.001	*F*_(3, 176)_ = 46.430, *p* < 0.001	*F* (2, 177) = 49.355, *p* < 0.001	*F* (3, 176) = 71.018, *p* < 0.001

^*^ *p* < 0.05, ^**^ *p* < 0.01, ^***^ *p* < 0.001.

In summary, field intervention results demonstrated that the combined implementation of leaders' mindfulness in communication training and employees' mindfulness training was superior to the other two approaches and could reduce employees' knowledge hiding behavior by enhancing their perceived organizational support.

## 6 Conclusions and discussion

### 6.1 Research findings

By integrating a moderated serial mediation model with a controlled field intervention, this study examined how and when leaders' mindfulness in communication reduces employees' knowledge hiding behavior. Across two complementary studies, the findings provide convergent evidence that mindful communication is not merely a positive interpersonal style but a meaningful managerial behavior that can reshape employees' psychological evaluations of the organization and reduce defensive knowledge behaviors.

First, leaders' mindfulness in communication was found to be negatively associated with employees' knowledge hiding behavior. Study 1 showed a significant negative relationship between leaders' mindfulness in communication and knowledge hiding, and Study 2 provided additional intervention-based evidence that employees whose leaders received mindfulness in communication training reported lower levels of knowledge hiding than those in the control group This finding suggests that knowledge hiding is not only influenced by formal rules, incentives, or broad leadership styles, but can also be reduced through specific communication behaviors that are observable and trainable. When leaders listen attentively, respond empathically, and communicate without premature judgment, employees may perceive lower relational risk and become less motivated to protect knowledge defensively.

Second, perceived organizational support, organizational justice, and interpersonal trust constituted the core psychological mechanisms linking leaders' mindfulness in communication to knowledge hiding. Specifically, leaders' mindfulness in communication enhanced employees' perceived organizational support, which subsequently strengthened employees' perceptions of distributive justice, procedural justice, interactional justice, and interpersonal trust, thereby reducing knowledge hiding. This finding indicates that perceived organizational support is not the endpoint of leader communication effects; rather, it functions as a psychological hub through which employees translate supportive communication into more specific judgments about fairness and trustworthiness. In knowledge-sharing contexts, these judgments are particularly important because employees often decide whether to disclose or withhold knowledge by evaluating whether their contributions will be fairly recognized and whether others can be trusted not to misuse shared knowledge.

Third, employee mindfulness strengthened the positive relationship between leaders' mindfulness in communication and perceived organizational support. The results suggest that employees do not passively receive leader communication cues. Instead, their own mindfulness shapes how accurately and openly they process such cues. Employees with higher mindfulness are more likely to notice leaders' attentive listening, non-judgmental acceptance, and empathic responses, and to interpret these behaviors as genuine signals of organizational support. Study 2 further showed that the combined training of leaders and employees produced stronger reductions in knowledge hiding than leader training alone. This finding highlights the importance of alignment between the sender and receiver of mindful communication: leaders provide supportive social cues, whereas mindful employees are better able to perceive and internalize those cues.

Taken together, these findings suggest that reducing knowledge hiding requires more than encouraging employees to share knowledge. It requires creating a relational and interpretive context in which employees feel supported, treated fairly, and able to trust others. Leaders' mindfulness in communication contributes to this context by shaping the quality of interpersonal exchanges, while employee mindfulness strengthens the interpretation and internalization of supportive communication. Therefore, knowledge hiding can be reduced most effectively when organizations intervene at both the leadership communication level and the employee information-processing level.

### 6.2 Theoretical contributions

This study makes several theoretical contributions.

First, this study contributes to knowledge hiding research by shifting attention from relatively stable leadership styles and formal governance mechanisms to a specific, trainable communication behavior. Prior research has shown that leadership can influence employees' knowledge behaviors, but much of this work has emphasized broad leadership styles, such as ethical leadership or transformational leadership. Although such perspectives are valuable, they provide limited guidance for organizations seeking concrete and scalable interventions. By showing that leaders' mindfulness in communication can reduce employees' knowledge hiding behavior, this study identifies a more behaviorally specific route through which organizations can address defensive knowledge behavior. This extends knowledge hiding research by demonstrating that employees' willingness to disclose knowledge is shaped not only by organizational policies or general leadership orientations, but also by the micro-level quality of leader–employee communication.

Second, this study extends workplace mindfulness research by examining mindfulness as a cross-level and interactive phenomenon. Existing workplace mindfulness studies often focus either on leader mindfulness or employee mindfulness separately. In contrast, the present study examines how leaders' mindfulness in communication and employee mindfulness jointly influence knowledge hiding. The findings show that leader mindfulness in communication provides supportive social cues, whereas employee mindfulness determines how strongly these cues are perceived and transformed into perceived organizational support. Notably, Study 2 also suggested a potential spillover effect: employees in the leader-training-only condition showed a marginal increase in employee mindfulness, even though they did not receive direct mindfulness training. Although this finding should be interpreted cautiously, it suggests that leaders' mindful communication may diffuse downward through social modeling and improved relational quality. Thus, this study enriches workplace mindfulness research by showing that mindfulness may operate not only as an individual psychological capacity but also as a relational process embedded in leader–employee interaction.

Third, this study advances social exchange theory by specifying the exchange-based mechanism through which mindful communication reduces knowledge hiding. From a social exchange perspective, employees hide knowledge when they perceive that sharing may expose them to resource loss, unfair treatment, or relational exploitation. The present findings show that leaders' mindfulness in communication can interrupt this defensive logic by enhancing perceived organizational support and then strengthening justice perceptions and interpersonal trust. In this way, the study clarifies how a communication behavior becomes an exchange resource: mindful communication conveys respect, care, and psychological safety, which employees then convert into judgments that the organization is supportive, fair, and trustworthy. This extends social exchange theory by identifying perceived organizational support as a central bridge connecting leader communication with employees' defensive knowledge behavior.

Fourth, this study strengthens the application of social information processing theory to knowledge hiding research. The findings show that the effect of leaders' mindfulness in communication depends partly on employees' mindfulness, indicating that employees differ in how they attend to and interpret leader communication cues. This contributes to social information processing theory by showing that workplace cues do not influence behavior automatically; rather, their effects depend on employees' cognitive and attentional capacities. In the present model, leaders' mindfulness in communication functions as a social cue, employee mindfulness shapes cue interpretation, perceived organizational support reflects the resulting psychological evaluation, and reduced knowledge hiding represents the behavioral response. By integrating social exchange theory and social information processing theory, this study explains both why supportive communication reduces knowledge hiding and when this process becomes stronger.

### 6.3 Practical implication

The findings also provide several implications for management practice.

First, organizations should incorporate mindfulness in communication into leadership development programs. The intervention results indicate that training leaders in attentive listening, non-judgmental acceptance, and empathic responding can reduce employees' knowledge hiding behavior. Compared with attempting to reshape an entire leadership style, communication-focused mindfulness training is more specific and easier to implement. Organizations can include modules on mindful listening, emotion regulation during difficult conversations, and non-defensive responses to employee concerns in leadership training programs. These practices can help leaders convey support and psychological safety in daily interactions, thereby reducing employees' perceived need to protect knowledge defensively.

Second, organizations should recognize perceived organizational support as a key psychological target in knowledge management. The findings suggest that leaders' mindful communication reduces knowledge hiding partly because it enhances employees' perceived organizational support, which then strengthens justice perceptions and interpersonal trust. Therefore, organizations should not rely solely on knowledge-sharing platforms or incentive systems. They should also ensure that employees feel their contributions are valued, their interests are protected, and their knowledge-sharing efforts are recognized. For example, managers can provide timely feedback on employees' knowledge contributions, publicly acknowledge helpful knowledge sharing, and ensure that knowledge contributors receive fair credit in evaluation and promotion processes.

Third, organizations should cultivate fairness and trust as downstream conditions for knowledge sharing. The results indicate that organizational justice and interpersonal trust are important mechanisms through which perceived organizational support reduces knowledge hiding. This means that employees may still hide knowledge if they believe that rewards are unfair, procedures are unclear, or colleagues may misuse shared information. Organizations should therefore strengthen distributive justice by linking knowledge contributions to fair recognition, procedural justice by establishing transparent knowledge-sharing rules, and interactional justice by encouraging respectful communication. At the same time, team leaders should build interpersonal trust by clarifying norms of reciprocity, discouraging opportunistic use of others' knowledge, and creating psychologically safe collaboration routines.

Fourth, employee mindfulness training can be used as a complementary intervention when organizational conditions permit. Study 2 showed that training both leaders and employees produced stronger reductions in knowledge hiding than training leaders alone. This suggests that leadership training may be more effective when employees also develop the attentional and emotional capacities needed to perceive and internalize supportive communication. Organizations can therefore offer brief mindfulness programs, reflective exercises, or communication workshops to help employees regulate defensive reactions, process social cues more accurately, and engage more openly in knowledge collaboration. Such programs should be framed not as individual therapy but as capacity-building interventions that support collaboration, communication, and knowledge flow.

### 6.4 Limitations and future research

First, the participants in this study were predominantly from technology companies in China. Although some work teams had an international background, the findings still need to be further validated in organizations across different industries and cultural contexts. Future research is encouraged to collaborate with cross-cultural research teams to examine the generalizability of these findings in diverse industrial and cultural settings. Second, although we extended the intervention to a 4 week structured mindfulness training program to enhance intervention strength and stability, whether longer or periodically repeated training produces more durable behavioral changes remains an open question. Future research, with more substantial funding, could adopt longitudinal or periodic training designs to examine the dynamic evolution and persistence of mindfulness training effects over time.

## Data Availability

The raw data supporting the conclusions of this article will be made available by the authors, without undue reservation.
